# MEG event-related desynchronization and synchronization deficits during basic somatosensory processing in individuals with ADHD

**DOI:** 10.1186/1744-9081-4-8

**Published:** 2008-02-12

**Authors:** Colleen Dockstader, William Gaetz, Douglas Cheyne, Frank Wang, F Xavier Castellanos, Rosemary Tannock

**Affiliations:** 1 Neurosciences and Mental Health Program, The Hospital for Sick Children, Toronto, Canada; 2 Department of Diagnostic Imaging, The Hospital for Sick Children, Toronto, Canada; 3 Child Study Center, New York University, New York, USA; 4 Human Development & Applied Psychology, Ontario Institute for Studies in Education, Toronto, Canada

## Abstract

**Background:**

Attention-Deficit/Hyperactivity Disorder (ADHD) is a prevalent, complex disorder which is characterized by symptoms of inattention, hyperactivity, and impulsivity. Convergent evidence from neurobiological studies of ADHD identifies dysfunction in fronto-striatal-cerebellar circuitry as the source of behavioural deficits. Recent studies have shown that regions governing basic sensory processing, such as the somatosensory cortex, show abnormalities in those with ADHD suggesting that these processes may also be compromised.

**Methods:**

We used event-related magnetoencephalography (MEG) to examine patterns of cortical rhythms in the primary (SI) and secondary (SII) somatosensory cortices in response to median nerve stimulation, in 9 adults with ADHD and 10 healthy controls. Stimuli were brief (0.2 ms) non-painful electrical pulses presented to the median nerve in two counterbalanced conditions: unpredictable and predictable stimulus presentation. We measured changes in strength, synchronicity, and frequency of cortical rhythms.

**Results:**

Healthy comparison group showed strong event-related desynchrony and synchrony in SI and SII. By contrast, those with ADHD showed significantly weaker event-related desynchrony and event-related synchrony in the alpha (8–12 Hz) and beta (15–30 Hz) bands, respectively. This was most striking during random presentation of median nerve stimulation. Adults with ADHD showed significantly shorter duration of beta rebound in both SI and SII except for when the onset of the stimulus event could be predicted. In this case, the rhythmicity of SI (but not SII) in the ADHD group did not differ from that of controls.

**Conclusion:**

Our findings suggest that somatosensory processing is altered in individuals with ADHD. MEG constitutes a promising approach to profiling patterns of neural activity during the processing of sensory input (e.g., detection of a tactile stimulus, stimulus predictability) and facilitating our understanding of how basic sensory processing may underlie and/or be influenced by more complex neural networks involved in higher order processing.

## Background

Attention-Deficit/Hyperactivity Disorder (ADHD) is an impairing neurodevelopmental disorder that remains inadequately understood. Along with the observable behavioral symptoms of inattention and hyperactivity/impulsivity, there is robust evidence of structural, functional, and neurochemical brain differences in ADHD [1-3] particularly in regions involved in vital executive functions (EFs) that regulate the ability to identify, extract, and interpret what is relevant for executing the correct response, as well as monitoring, inhibiting, and changing the prepotent response as needed [4,5]. The pathophysiology of ADHD remains unclear, although converging evidence suggests that alterations in brain structure, function, and physiology likely arise from an interaction of genetic and environmental causes and experience [5-8]. For example, structurally, prominent volumetric decreases are evident in the posterior-inferior lobules of the cerebellar vermis in both male and female children with ADHD [9-12]. There are decreases in prefrontal volume, particularly the right prefrontal cortex [9,13]. Also reported are regional differences in cerebral blood flow in the cerebellum, striatum [14] and prefrontal cortex (PFC) [15]. Moreover, differences in baseline oscillatory activity between those with ADHD and controls have been observed in frontal regions, particularly the PFC [16,17]. Consistent with the neuroimaging findings, psychological research indicates clearly that subtle but impairing problems in EFs are correlates of ADHD, regardless of gender or age [18].

While the majority of ADHD research focuses on deficits in EF, it is apparent that not all individuals with ADHD have EF deficits [18,19] and that not all neuropsychological difficulties can be explained by EF theory alone [20]. Moreover, EF tasks in which individuals with ADHD do show deficits often include processing and responding to simple sensory stimuli that vary in predictability. This suggests that deficits in anticipatory or perceptual processing of simple stimuli could also contribute to impairments on tasks that assess higher-order functions. Accordingly, an important goal of ADHD research is to address not only the concept of multiple *forms *of impairment but also of multiple *sources *of impairment. Emerging evidence not only shows abnormalities in neural regions governing higher order function but also in regions governing basic function such as somatosensory cortex [21-24], motor cortex [21,25,26] and visual cortex [27]. Although people with ADHD have shown behavioural deficits in responding to simple stimuli during sensorimotor tasks [28-30], methodological shortcomings in the limited studies available have precluded an adequate understanding of the role of neural networks in processing predictable and non-predictable stimuli in ADHD. Specifically, existing studies have relied almost exclusively on behavioural measures (i.e., accuracy, reaction time), which cannot assess moment-by-moment activities that are driving these processes on the order of milliseconds.

Our aim was to examine basic sensory processing of predictable and non-predictable stimuli in those with ADHD using magnetoencephalography (MEG), a non-invasive functional neuroimaging technique that records neural activity on the order of a millisecond. This high temporal resolution combined with novel source reconstruction techniques capable of mm spatial resolution makes MEG an optimal technique for capturing spatial and temporal information during sensory processing for which the time scale is on the order of milliseconds. MEG studies of the human somatosensory system using median nerve stimulation have shown that only the contralateral primary somatosensory cortex (SI) responds to unilateral tactile information whereas bilateral secondary somatosensory cortices (SII) show activity in response to unilateral stimulation [31,32]. The earliest somatosensory activity occurs at approximately 20 ms post-stimulation in SI just caudal to the central sulcus in the corresponding topographical location. Subsequent somatosensory activation occurs in the bilateral parietal opercula located in the dorsal regions of the lateral sulci [32-34]. Source activity in SI and SII, following median nerve stimulation, is composed of both alpha and beta cortical rhythms [35]. In association with MEG, median nerve stimulation has been used to examine evoked responses to somatosensory stimuli in order to examine somatosensory cortical function [31,36,37] and ascending pathways from the peripheral receptors to the spinal cord, brainstem, thalamus, and cortex [38]. This technique has also been used to examine physical and cognitive impairments in individuals with Alzheimer's [39], stroke patients [40], and infantile autism [41], for example. Using MEG, we investigated the oscillatory changes during somatosensory activation in adults with and without ADHD.

The general assumption of cortical oscillations is that populations of neurons exist in varying states of synchrony as they respond to externally or internally generated events. Event-related desynchrony (ERD) and event-related synchrony (ERS) phenomena are thought to represent decreases and increases, respectively, in synchronization within a specific frequency range in relation to an event [42]. Previous MEG studies of cortical activity following median nerve stimulation in healthy adults report brief suppression of mu (an alpha wave variant oscillating at approximately 10 Hz) and beta (15–30 Hz) cortical activity in primary and secondary somatosensory cortex (ERD) followed by a marked increase in beta band activity above baseline (late-ERS, known as *beta rebound*) [42]. Basic or complex sensory processing requires a dynamic interaction between groups of neurons oscillating at particular frequencies and differing degrees of coupling. Oscillations in the alpha and beta bands are of particular interest in ADHD research as these frequencies are thought to mediate perception [43,44] and attention [45-47]. To our knowledge, MEG has not yet been used to investigate changes in SI alpha or beta oscillations in individuals with ADHD. Accordingly, our aim was to characterize ERD and ERS in the alpha and beta bands in SI and SII in response to randomly and predictably presented electrical stimulation of the median nerve in adults with and without ADHD. Comparison of random versus predicted median nerve stimulation is a novel approach to determine whether basic somatosensory processing differs between those with ADHD and healthy controls and if stimulus predictability differentially influences somatosensory processing in those with ADHD compared to controls.

The neural basis of predictive responding to the *absence *of a stimulus in both SI and SII will be described in a subsequent report.

## Methods

### Participants

We studied nine adults (4 females/5 males) with a diagnosis of ADHD (mean age: 34.6 +/- 3.28 years) and ten healthy age-matched controls (4 females/6 males; mean age of 34.13 +/- 4.6 years). All were right-handed. Adults with ADHD were recruited from an outpatient neuropsychiatry clinic in a mental health centre in a large metropolitan city. All had completed the same comprehensive clinical diagnostic assessment including: a clinical diagnostic interview and various self-report rating scales including the Conners Scales [48]; Wender Utah Rating Scale [49], Brown Attention Deficit Disorder Scales (Brown, 1996); and Adult Self-Report Scale [50]. Healthy adult volunteers were recruited by means of advertisements placed in the same institution and in other community organizations. All participants completed a telephone-based Intake Screening Questionnaire (screens for psychopathology and education level) and the Adult ADHD Self-Report Scale [51] at the time of participation to estimate current levels of ADHD symptomatology. Participants were excluded if they wore orthodontic braces, had any non-removable metal, or had a diagnosis of psychosis, neurological disorder, or uncorrected sensory impairments.

### MEG Recordings

A whole-head 151 channel MEG system (VSM MedTech Ltd, Vancouver, Canada) was used to measure somatosensory evoked fields. Participants lay in a supine position in a magnetically shielded room with their head resting in the MEG helmet. The MEG signals were bandpass filtered at 0.3 – 300 Hz and recorded at a 1250 Hz sampling rate. Head position in relation to the MEG sensors was determined by measuring the magnetic field generated by 3 fiducial reference coils just before and after each experimental session. T_1_-weighted structural magnetic resonance images (MRI) (axial 3D spoiled gradient echo sequence) were obtained for each participant using a 1.5 Tesla Signal Advantage system (GE Medical Systems, Milwaukee, USA). During MRI data acquisition, 3 radiographic markers were positioned on the same anatomical landmarks as the fiduciary coils to allow coregistration accuracy of the MEG and MRI data. Single equivalent current dipole (ECD) models were also fit to the N20m median nerve responses in order to confirm coregistration accuracy.

### Experimental Paradigm

Individuals were asked to lie comfortably on a bed in the MEG room and relax. Stimuli were non-painful, current pulses of 0.2 ms duration, presentation rate of 0.5 Hz (ISI: 2000 ms between onset of each stimulus), just above motor threshold (eliciting a small, passive, thumb twitch) applied cutaneously to the right median nerve. Somatosensory stimuli were presented in two counterbalanced conditions: *a*) Predicted Stimulus Pattern and *b*) Random Stimulus Pattern. In the Predicted Pattern, stimuli occurred in trains of four followed by a long break before the next train (4000 ms) giving 332 stimulus events (83 trains) and 83 breaks in between trains. In the Random Pattern, stimuli and long breaks (4000 ms) were randomly dispersed throughout a 415 event trial (totaling 332 stimuli and 83 breaks). Each condition was 12 minutes in length. Participants were naïve as to the specific patterns that were presented. Upon completion of both stimulus conditions, each participant was asked if they recognized a presentation pattern or not in each of the paradigms.

This research was conducted in compliance with the Helsinki Declaration and approved by the Research Ethics Board at The Hospital for Sick Children, Toronto, Canada, File Number 1000010728.

### Data analyses

Neural activities during the experiments were analyzed with respect to brain location, latency, and frequency to determine spatiotemporal profiles of event-related activity time-locked to stimulus presentation. Initial spatial analyses were performed using a novel application of a minimum-variance beamformer algorithm (synthetic aperture magnetometry: SAM) [52-54]. In order to map the median nerve initial response we created SAM differential images by subtracting control periods (-200 to 0 ms prior to stimulus or gap onset) from active periods (0 to 200 ms after stimulus or gap onset) and filtering the data from 3 – 50 Hz. This resulted in high resolution (2 mm) three-dimensional differential images which were time-locked to median nerve stimulation and averaged over time to identify peak activation sites in the brain during the active period relative to baseline. Grand averaged localizations of regional activity peaks for each group were determined by warping SAM images to a template brain and averaging across subjects using Statistical Parametric Mapping software [55]. Source activity was overlaid on the template brain and imaged using mri3dX software [56].

We then computed the single trial output of the spatial filters ('virtual sensors') for peak locations of source activity in the SAM images displaying millisecond changes in source power. Time-frequency response (TFR) plots were constructed from the virtual sensor data using a wavelet-based technique which demonstrates both phase-locked and non-phase-locked changes in power at different frequencies over time relative to the baseline period (-100 ms to 0 ms prior to stimulus onset).

Following TFR results we wished to examine group differences for specific frequency sets. Selected bandwidths were averaged across subjects, demonstrating the time course of averaged group response amplitude for a chosen frequency set. Regions on the line graphs were highlighted wherever standard errors did not overlap between controls and those with ADHD in order to exemplify bandwidths where the two groups diverged significantly over time. To determine statistical differences between groups and conditions for each separate time-frequency value we used a permutation program that extracted the normalized, source power value for each time-frequency bin. Individual data were subject to 1000 permutations and then collapsed across participants within a specific group and experimental condition to derive a mean value which could then be statistically compared between groups or conditions. The group mean difference for each pixel was plotted (i.e. – control group data minus ADHD group data for SI random condition) and subsequently thresholded so that only statistically significant differences remained, being expressed as a P-Value plot. Multiple comparison corrections (such as a Bonferroni correction) were *not *made to the data as each TFR point was not independent.

## Results

### Primary somatosensory cortex (SI)

#### Random stimulus presentation

Neural activity captured from each of the 151 MEG channels was averaged over the total number of trials in which a stimulus was presented, and then spatial analyses were performed using SAM to create differential images that represented changes from baseline in neural activity.

**Figure 1:**
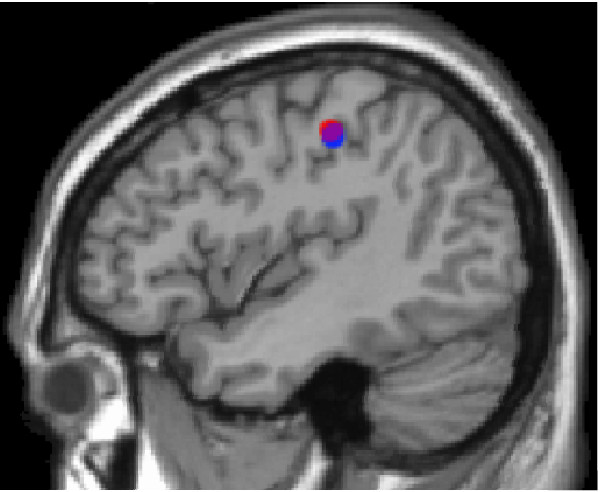
Grand Mean Contralateral SI localization for Control (blue) and ADHD (red) Based on SAM Differential Analyses.

SAM analysis indicated that for individuals in both the comparison and ADHD groups, the control period showed little or no somatosensory activation with average peak values being approximately 5% of active period values (for average peak values during control and active periods see Additional files 1 &2 for control and ADHD groups, respectively). During the active period, maximal peak activity was localized specifically to the hand area, area 3b, in the contralateral primary somatosensory cortex (SI) (Figure 1). The virtual sensor of each individual's contralateral SI location identified that for the control group the grand-averaged maximal activity occurred at 22.02 ms +/- 0.74 SEM post-stimulus and at 21.78 ms +/- 0.86 SEM for the ADHD group. There was no statistical difference of response time [t(17) = 0.303, *p *> 0.05].

**Figure 2:**
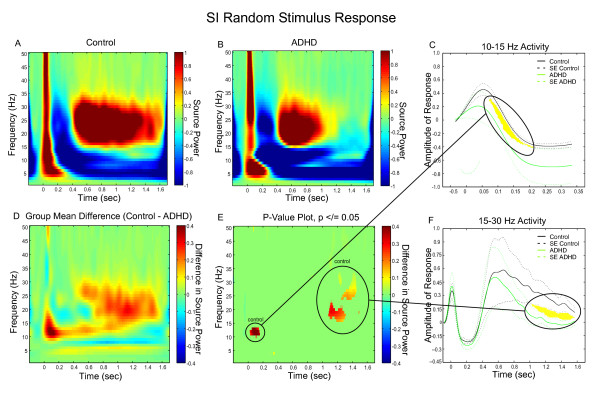
**SI Group Differences in Frequency and Power During Random Presentation of a Somatosensory Stimulus**. **(A) **Grand Mean TFR of the individual, virtual channel, spatially-filtered single trials for control subjects. In both control subjects and subjects with ADHD the plot was baselined using the average spectral energy observed in the pre-stimulus period (-100 – 0 ms). **(B) **Grand Mean TFR of the individual, virtual channel, spatially-filtered single trials for subjects with ADHD. **(C) **Group mean differences of the group TFRs. **(D) **Statistically significant values remaining once group differences were thresholded to p </= 0.05. **(E) **Divergence of early response to the stimulus in controls and ADHD. **(F) **Divergence of power in beta rebound in the latter portion of the trial between controls and ADHD.

Time-Frequency Representation (TFR) plots clearly demonstrated that there were three distinct phases of rhythmic changes in the somatosensory cortex. These occurred in both primary and secondary cortices (SI and SII, respectively) in both controls and those with ADHD; (i) early-ERS (approximately 20 to 200 ms post-stimulus); (ii) ERD (approximately 200 to 400 ms post-stimulus); and (iii) late-ERS, known as *beta rebound *(approximately 400+ ms post-stimulus). In the control group, the early ERS in SI was a broad-frequency increase in power occurring approximately 20 to 200 ms post-stimulus. This immediate response was followed by a broad-frequency ERD; a transitory suppression of source power below baseline that occurred from 200 to 400 ms post-stimulus. Following the suppression, the final phase of activity demonstrated a rebound of ERS specific to the beta band that began approximately 400 ms after median nerve stimulation and lasted for about 1200 ms (Figure 2A). Our findings are consistent with previous research showing that beta rebound begins between 250 ms [57,58] and 500 ms [58] following median nerve stimulation.

In the ADHD group the early SI ERS, occurring approximately 20 to 200 ms post-stimulus, was characterized by strong early ERS in the lower bandwidths (theta and low alpha) and high beta band (25+ Hz), with more moderate activity in the midrange (10 to 22 Hz) compared to baseline. ERD occurred across the spectrum of frequencies from 200 to 400 ms post-stimulus while beta rebound ERS commenced at approximately 400 ms but continued for only 600 ms which was considerably shorter in duration than controls (Figure 2B).

Group comparisons revealed that adults with ADHD showed substantially less power between 10 and 15 Hz during the early ERS, compared to the control group and substantially less power during beta rebound (Figure 2C). The group differences in neural response reached statistical significance between 30 and 180 ms and ranges from 11 to 14 Hz during the immediate response (Figure 2D). This is highlighted in the line graph (Figure 2E) where one can observe the considerable divergence of the two groups in amplitude of response across this particular frequency range over the first 200 ms of the trial (i.e., marked area in which there is no overlap of group standard error bars). Adults with ADHD also showed substantially less ERS than controls between 15 and 30 Hz in the latter half of the trial (1000+ ms) (also Figure 2D), indicating that individuals with ADHD experienced a significantly shorter beta rebound following a somatosensory event. The line graph demonstrates the power divergence of the entire beta bandwidths between groups during the rebound period (Figure 2F).

#### Predicted stimulus presentation

For controls, the temporal onset of the grand averaged SI response occurred at 21.30 ms +/- 0.94 SEM which did not statistically differ from the onset of the averaged control random response [t(9) = 0.79, *p *> 0.05]. Permutation analysis revealed that the grand averaged time-frequency responses for controls did not differ statistically between the random and predicted stimulus conditions (Additional file 3). Similarly, the grand averaged SI temporal onset of the ADHD group response occurred at 21.62 ms +/- 1.14 SEM which did not differ from their averaged random onset [*t*(8) = 0.198, *p *> 0.05] *or *from the control predicted temporal onset [t17) = -0.225, *p *> 0.05]. Interestingly, permutation analyses of the ADHD TFRs suggested a a slight within-group difference between conditions from 1200 to 1500 ms in the 17 to 25 Hz range (Additional file 4).

**Figure 3:**
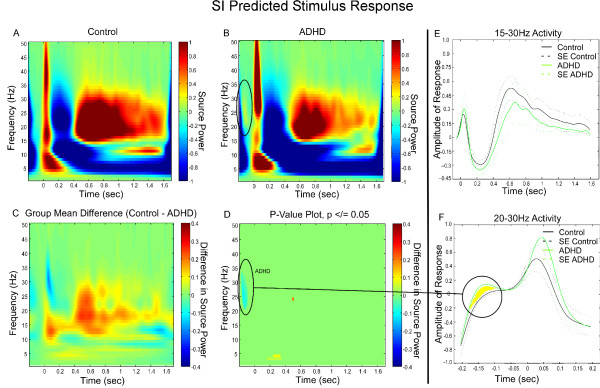
**SI Group Differences in Frequency and Power During Predicted Presentation of a Somatosensory Stimulus**. **(A) **Grand Mean TFR of the individual, virtual channel, spatially-filtered single trials for control subjects. In both control subjects and subjects with ADHD the plot was baselined using the average spectral energy observed in the pre-stimulus period (-100 – 0 ms). **(B) **Grand Mean TFR of the individual, virtual channel, spatially-filtered single trials for subjects with ADHD. **(C) **Group mean differences of the group TFRs. **(D) **Statistically significant values remaining once group differences were thresholded to p </= 0.05. **(E) **Groups show no divergence of early response to the stimulus in controls and ADHD. **(F) **Divergence of power in beta rebound in the pre-stimulus period between controls and ADHD.

In the control group, the TFR analysis of the early SI ERS revealed intense activity from 5 Hz to 50+ Hz followed by ERD and a strong beta rebound ERS (Figure 3A). Notably, SI mu rhythm showed ERD (150 ms to 900 ms) and beta rebound ERS (900 ms to 1600 ms) following tactile stimulation. The somatosensory region endogenously oscillates around this bandwidth [59,60] and SI mu synchronizations and desynchronizations have been identified consistently in response to median nerve stimulation [61].

The TFR analysis of the ADHD group showed a strong early ERS in the lower bandwidths (theta and low alpha) and high beta band (25 to 40 Hz), but little power in the midrange (10 to 22 Hz) followed by ERD and a rebound ERS composed of strong activation during the first half of the rebound period and reduced activation in the latter half (Figure 3B). Additionally, there was a subtle, transient beta response just prior to stimulus onset observed in the ADHD TFR grand mean (Figure 3B, circled).

Even though we observed some general group differences in power of the early ERS, ERD, and beta rebound ERS in the Predicted condition Group comparisons between controls and those with ADHD (Figure 3C) there were no statistical differences in power changes between groups during any of these phases (Figure 3D). The lack of a difference in beta activity during these phases is exemplified in the beta bandwidth line graph analyses (Figure 3E). However, examining SI activity in the pre-stimulus phase of the trial we observed a significant between-group difference that may suggest anticipatory neural responses in the ADHD group (also Figure 3D) when at approximately 170 ms prior to stimulus onset the two group responses began to diverge (Figure 3F) and the ADHD group showed an early increase in beta power that the control group did not.

### Secondary somatosensory cortex (SII)

SAM analyses showed prominent activation of secondary somatosensory cortices (SII) within the parietal operculum. Bilateral SII activation was observed with contralateral and ipsilateral activity profiles being very similar. For brevity, only contralateral SII information, the recipient of contralateral SI information, is reported.

#### Random stimulus presentation

**Figure 4:**
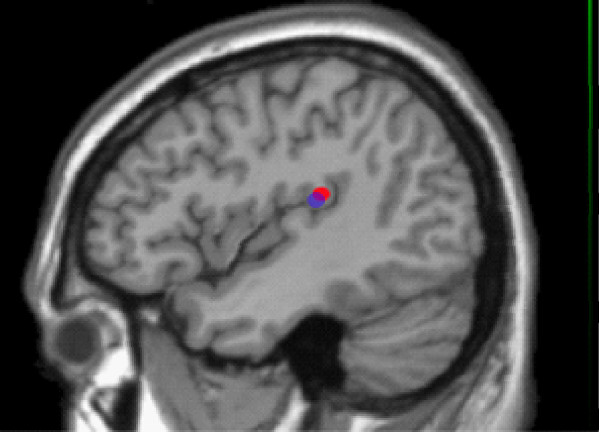
Grand Mean contralateral SII localization for control (blue) and ADHD (red) based on SAM differential analyses.

The control and ADHD groups both showed consistent, robust peak activity in SII (Figure 4). For controls, the grand-averaged virtual sensor identified the first activity peak at 41.20 ms +/- 2.43 SEM following a stimulus: for adults with ADHD it occurred at 43.15 ms +/- 3.16 SEM which did not statistically differ from the onset of the averaged control random response [t(17) = -0.803, *p *> 0.05].

**Figure 5:**
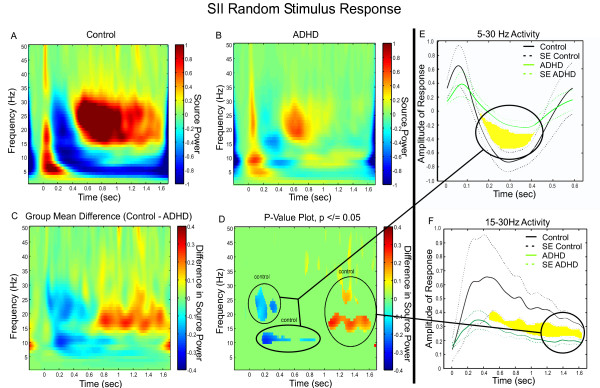
**SII Group Differences in Frequency and Power During Random Presentation of a Somatosensory Stimulus**. **(A) **Grand Mean TFR of the individual, virtual channel, spatially-filtered single trials for control subjects. In both control subjects and subjects with ADHD the plot was baselined using the average spectral energy observed in the pre-stimulus period (-100 – 0 ms). **(B) **Grand Mean TFR of the individual, virtual channel, spatially-filtered single trials for subjects with ADHD. **(C) **Group mean differences of the group TFRs. **(D) **Statistically significant values remaining once group differences were thresholded to p </= 0.05. **(E) **Divergence of the ERD response to the stimulus in controls and ADHD. **(F) **Divergence of power in beta rebound in the latter portion of the trial between controls and ADHD.

The grand-averaged TFR In the control group showed strong early ERS in the trial followed by strong ERD and beta rebound ERS activity similar to that observed in SI. The early ERS and the ERD was characterized by robust activity from 5 to 20 Hz, with the strongest activity oscillating around 7 Hz while frequencies 15 to 30 Hz contributed to the ensuing rebound (Figure 5A). By contrast, in the ADHD group the grand-averaged TFR showed a modest early ERS response characterized by clusters of source power oscillating at theta, alpha, and beta frequencies. Moreover, minimal ERD power was present in the alpha and low beta bandwidths and this was followed by a very brief, modest beta rebound ERS (Figure 5B).

Group comparisons revealed that overall, SII responding of adults with ADHD showed markedly less source power than that of controls (Figure 5C). These group differences were substantiated in the permutation analyses which revealed that adults with ADHD displayed significantly less ERD and significantly shorter beta rebound ERS across both alpha and beta activities (Figure 5D). The neural responses of the two groups diverged considerably during broad-spectrum ERD (Figure 5E) and during the ensuing beta rebound ERS (Figure 5F).

#### Predicted stimulus presentation

In the control group, the temporal onset of the grand averaged control SII response (41.85 ms +/- 1.20 SEM) did not statistically vary between random and predicted conditions [t(9) = -0.318, *p *> 0.05]. Grand averaged time-frequency responses did not statistically vary between the two conditions they experienced (Additional file 5). Correspondingly, the grand averaged SII temporal onset of response of adults with ADHD (40.38 ms +/- 1.97 SEM) did not vary in the predicted condition compared to the random condition [t(8) = 0.92, *p *> 0.05] *or *from the SII control predicted temporal onset [t(17) = 0.651, *p *> 0.05] Grand averaged time-frequency responses did not statistically vary between the two conditions the ADHD group experienced except for the period of ERD in which the SII response showed significantly more desynchrony in the alpha band (between 8 and 10 Hz) in the predicted condition than in the random one (Additional file 6).

**Figure 6:**
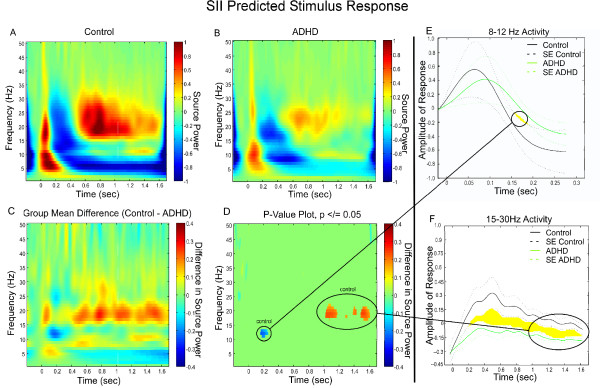
**SII Group Differences in Frequency and Power During Predicted Presentation of a Somatosensory Stimulus**. **(A) **Grand Mean TFR of the individual, virtual channel, spatially-filtered single trials for control subjects. In both control subjects and subjects with ADHD the plot was baselined using the average spectral energy observed in the pre-stimulus period (-100 – 0 ms). **(B) **Grand Mean TFR of the individual, virtual channel, spatially-filtered single trials for subjects with ADHD. **(C) **Group mean differences of the group TFRs. **(D) **Statistically significant values remaining once group differences were thresholded to p </= 0.05. **(E) **Divergence of the ERD response to the stimulus in controls and ADHD. **(F) **Divergence of power in beta rebound in the latter portion of the trial between controls and ADHD.

In the control group, SII exhibited strong early ERS from 5 Hz to 25 Hz followed by ERD and beta rebound ERS, consistent with the control Random SII response. Similar to the SI Predicted response and contrary to the SII Random response, the SII Predicted control response showed mu ERD followed by a rebound ERS late in the trial (Figure 6A).

In the ADHD group, the SII predicted response showed modest early ERS composed mostly of alpha and some beta oscillations followed by corresponding ERD and a small beta rebound ERS. Minor activation around 10 Hz from 1000 ms to 1600 ms suggests a faint mu rebound effect (Figure 6B).

Group comparisons revealed that, as in SII Random, the overall responding of adults with ADHD was considerably less than the controls (Figure 6C). The permutation analyses corroborated the findings revealing that individuals with ADHD displayed exhibited significantly less ERD and significantly shorter beta rebound ERS than the controls (Figure 6D). The two group responses diverged considerably within the ERD (alpha bandwidth) and beta rebound ERS phases (beta bandwidth) (Figure 6E &6F, respectively).

#### Debriefing interview

There were no group differences in their detection of stimulus patterns during median nerve stimulation: where no participants detecting a pattern during the random condition and all participants reported detecting a pattern during the predicted stimulus condition.

## Discussion

This study used MEG with median nerve stimulation to determine whether somatosensory processing was altered in adult ADHD. We measured frequency specific changes in evoked spatiotemporal patterns of neural activation in response to non-painful electrical stimuli in adults with and without ADHD. Major findings included a marked reduction in the duration of beta rebound in the ADHD group compared to controls in both SI and SII. Beta rebound is a post-stimulus beta phenomenon which commences approximately 400 – 600 ms after median nerve stimulation. Additionally, the ADHD group showed a substantial decrease in SII alpha and beta power during ERD (decreases in power of cortical oscillations below baseline) and ERS (increases in power of cortical oscillations above baseline).

When the stimuli were randomly presented, the ADHD group showed reduced SI ERS power during the immediate N20m response and a significantly shorter SI beta rebound than the controls. This suggests that incoming somatosensory information is less well-characterized at a basic neural level in those with ADHD. Irrespective of whether stimuli were randomly *or *predictably presented, the ADHD group showed substantive power decreases in SII alpha and beta ERD and SII beta rebound ERS relative to controls as well as a significantly shorter SII beta rebound. From SI, somatosensory information is thought to project to SII, where stimulus information is integrated and contextualized [62,63]. Without sufficient consolidation at SI the deficit may become even more profound as the information is volleyed to the higher processing region of SII. This would explain the marked reduction in SII ERD and ERS in the ADHD group.

To our knowledge this is the first demonstration of reduced duration of somatosensory evoked beta rebound in a clinical population. Little is known regarding the functional significance of the beta rebound response. Historically, beta rebound was thought to be an epiphenomenon that originated in the motor cortex in response to volitional movement [64]. More recent MEG recordings show that beta rebound also occurs in somatosensory cortex and can be initiated by a tactile stimulus, in the absence of volitional movement [47]. Moreover, attending to a stimulus can suppress beta rebound relative to that occurring when the stimulus is intentionally ignored [47]. Both movement imagery [65] and observation [66] have been found to suppress the rebound effect. Collectively, these findings suggest that beta rebound can be associated with cognitive state.

Further evidence supports the notion that beta rebound plays a significant role in cortical inhibition of neural regions unrelated to current task performance [42]. For instance, Chen et al [67] showed that the brain is less responsive to transcranial magnetic stimulation during the period of beta rebound following median nerve stimulation. If cortical inhibition is indexed by levels of beta activity then it might be argued that the lower levels of beta activity in individuals with ADHD reflect increases in cortical activity. Functional imaging studies indicate that individuals with ADHD activate more widespread brain regions than controls during task performance (review: [68]).

To our knowledge, this is the first application of MEG to investigate changes in somatosensory alpha or beta power in individuals with ADHD. Here we demonstrate that adults with ADHD showed less changes in source power in the alpha and beta bands overall in response to a somatosensory stimulus. Correspondingly, reduced alpha and beta powers have consistently been associated with ADHD EEG profiles (review: [69]). Intriguingly, when the adults in the ADHD group were able to predict the onset of an impending event, their SI response to a stimulus did not differ statistically from controls. It may be that the small sample size precluded our ability to detect an underlying effect, as the group mean time frequency plots for the SI Predicted condition in the ADHD and control groups appear different, however these differences did not reach statistical significance. Alternatively, it may be the case that, when a stimulus is predictable, individuals with ADHD are able to recruit additional resources to facilitate somatosensory processing, thereby concealing underlying primary deficits. A similar effect has been observed in individuals with obsessive-compulsive disorder whose behavioural performance was the same as controls in a visual working memory task [70]. This occurred in spite of the fact that these patients had significantly weaker desynchrony in the alpha band in response to a visual stimulus during the task with a distracter present but not when the distracter was absent [70].

Our findings support the notion that cortical oscillations are altered during somatosensory processing in those with ADHD. It is possible that impaired somatosensory processing may impede sensorimotor development, as has been found in a substantial proportion of children with ADHD [71-74]. Our data may explain, in part, why individuals with ADHD perform poorly on tasks that require somatosensory feedback such as externally-paced finger-tapping tasks [28,30,75,76] especially when the tasks require that tactile information be integrated in higher processing regions. Alternatively, it is possible that deficits in attention or executive functions may exert top-down influences on somatosensory processing in the ADHD group.

Our study is limited by the fact that we were unable to investigate effects of gender, comorbidity, or treatment history as the sample of adults with ADHD used in this study was small and heterogeneous, with variation in age, comorbidity, and/or medication (although medication was stopped for at least 24 hours prior to the study). Additionally, right hemisphere SI activity was not investigated as median nerve stimulation was only delivered to the dominant arm. In spite of these limitations, several findings reach statistical significance, emphasizing the powerful nature of the differences in somatosensory processing between the two groups. Future studies will investigate the effects of gender, comorbidity, and medication, as well as the activity of the right SI in response to a contralateral stimulus. Future steps to examine the cortical activity in regions that are in communication with the somatosensory cortex are necessary goals to further elucidate differences in basic processing in individuals with ADHD.

In summary, this study revealed several novel observations regarding somatosensory activity in an ADHD population. It is the first to profile somatosensory ERS and ERD in ADHD and the first to show that beta rebound is not a uniform phenomenon but one that can be modified in the presence of a psychiatric disorder. Profiling impaired cortical rhythms in response to basic sensory processing in ADHD will provide a more in depth understanding of the breadth of deficits in individuals with ADHD and aid in reconstructing the conceptualization and clinical understanding of ADHD.

## Competing interests

The author(s) declare that they have no competing interests.

## Authors' contributions

CD developed the design of the study, carried out MEG recordings (subject testing), statistical analyses, and drafted the manuscript. WG participated in the design of the study, contributed to the interpretation of the results. DC participated in the design of the study and contributed to the development of the source analysis methods and interpretation of the results. FW carried out MEG recordings, statistical analyses, and computer programming. FXC contributed to the interpretation of the results. RT participated in the overall conceptualization and supervision of project, including the design and interpretation of the results, All authors read, contributed to, and approved the final manuscript.

## Supplementary Material

Additional file 1 Example of a control subject's SAM peak locations and values during control (SAM peak value = 2.0) and active (SAM peak value = 16.3) states in somatosensory cortex Click here for file

Additional file 2 Example of an ADHD subject's SAM peak locations and values during control (SAM = 1.3) and active (SAM peak value = 15.0) states in somatosensory cortex Click here for file

Additional file 3 **Control SI Frequency and Power Dynamics During Predicted versus Random Presentation of a Somatosensory Stimulus (A)**. Grand Mean TFR of the individual, virtual channel, spatially-filtered single trials for control subjects during Predicted presentation of a stimulus. The plot was baselined using the average spectral energy observed in the pre-stimulus period (-100 – 0 ms). **(B) **Grand Mean TFR of the individual, virtual channel, spatially-filtered single trials for control subjects during Random presentation of a stimulus. **(C) **Mean TFR differences between conditions. **(D) **Statistically significant values remaining once condition differences were thresholded to p </= 0.05. Click here for file

Additional file 4 **ADHD SI Frequency and Power Dynamics During Predicted versus Random Presentation of a Somatosensory Stimulus (A)**. Grand Mean TFR of the individual, virtual channel, spatially-filtered single trials for subjects with ADHD during Predicted presentation of a stimulus. The plot was baselined using the average spectral energy observed in the pre-stimulus period (-100 – 0 ms). **(B) **Grand Mean TFR of the individual, virtual channel, spatially-filtered single trials for subjects with ADHD during Random presentation of a stimulus. **(C) **Mean TFR differences between conditions. **(D) **Statistically significant values remaining once condition differences were thresholded to p </= 0.05. Click here for file

Additional file 5 **Control SII Frequency and Power Dynamics During Predicted versus Random Presentation of a Somatosensory Stimulus (A)**. Grand Mean TFR of the individual, virtual channel, spatially-filtered single trials for control subjects during Predicted presentation of a stimulus. The plot was baselined using the average spectral energy observed in the pre-stimulus period (-100 – 0 ms). **(B) **Grand Mean TFR of the individual, virtual channel, spatially-filtered single trials for control subjects during Random presentation of a stimulus. **(C) **Mean TFR differences between conditions. **(D) **Statistically significant values remaining once condition differences were thresholded to p </= 0.05. Click here for file

Additional file 6 **ADHD SII Frequency and Power Dynamics During Predicted versus Random Presentation of a Somatosensory Stimulus (A)**. Grand Mean TFR of the individual, virtual channel, spatially-filtered single trials for subjects with ADHD during Predicted presentation of a stimulus. The plot was baselined using the average spectral energy observed in the pre-stimulus period (-100 – 0 ms). **(B) **Grand Mean TFR of the individual, virtual channel, spatially-filtered single trials for subjects with ADHD during Random presentation of a stimulus. **(C) **Mean TFR differences between conditions. **(D) **Statistically significant values remaining once condition differences were thresholded to p </= 0.05. Click here for file
