# Supplementary figures and images for: MEG event-related desynchronization and synchronization deficits during basic somatosensory processing in individuals with ADHD
Source: Behav Brain Funct. 2008 Feb 12;4:8. doi: 10.1186/1744-9081-4-8 (PMC2266931; doi:10.1186/1744-9081-4-8)

## Control subject

Control Period: -0.2 sec to 0.0 sec

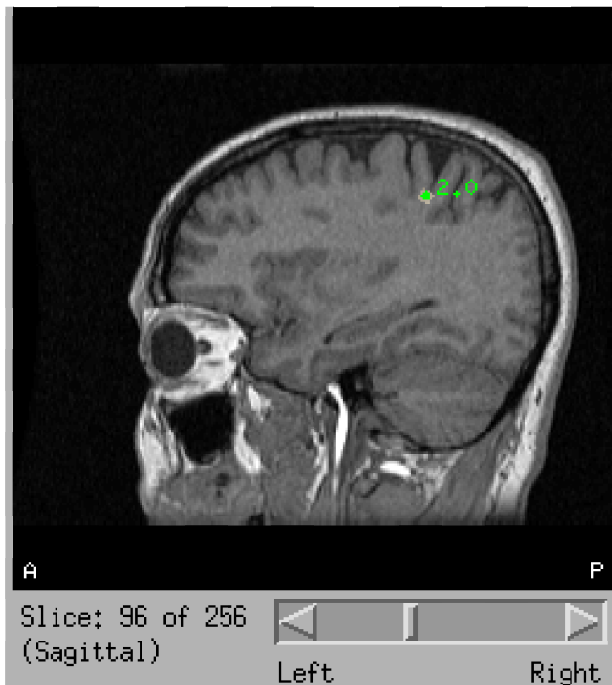

Active Period: 0.0 sec to 0.2 sec

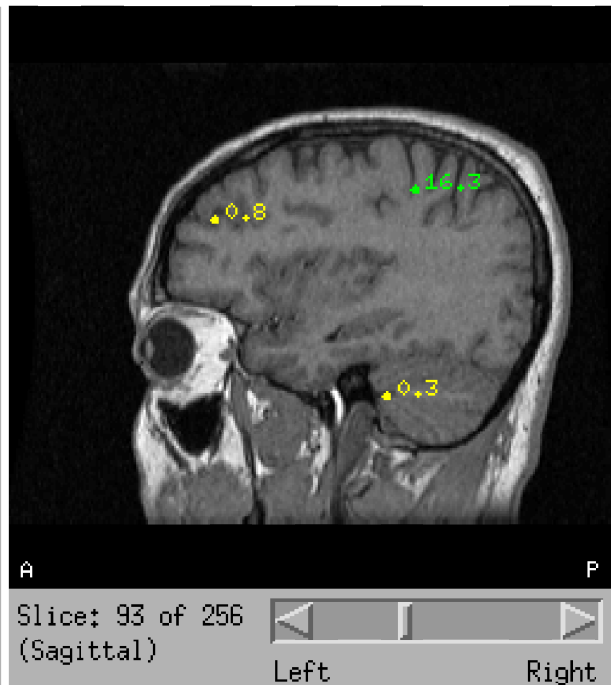

Supplement: Additional file 1 — Example of a control subject's SAM peak locations and values during control (SAM peak value = 2.0) and active (SAM peak value = 16.3) states in somatosensory cortex [file 1744-9081-4-8-S1.pdf]

# SI Control Stimulus Response

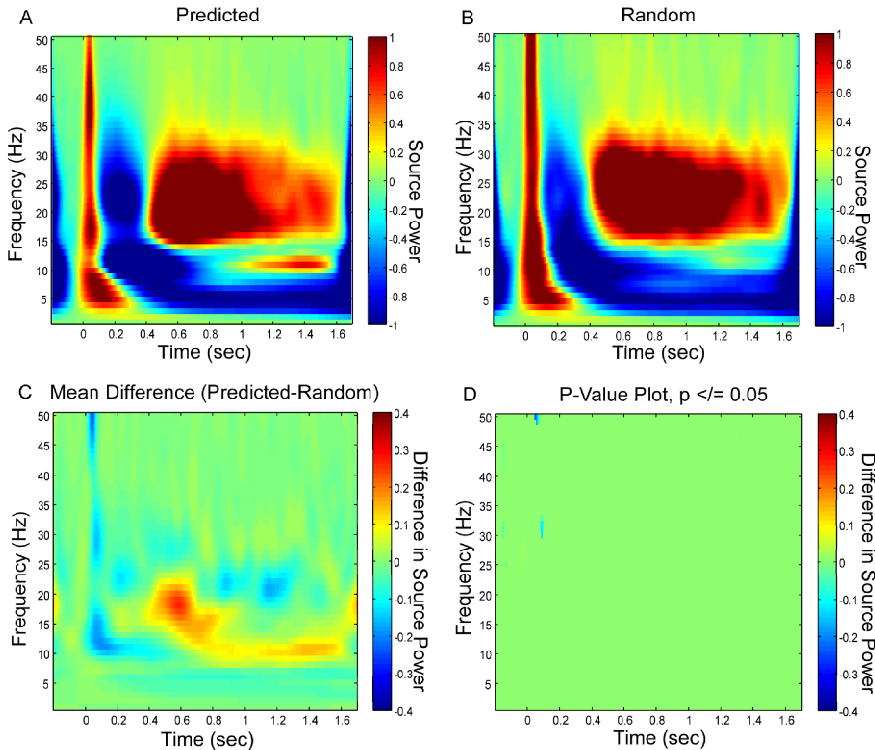

Supplement: Additional file 3 — Control SI Frequency and Power Dynamics During Predicted versus Random Presentation of a Somatosensory Stimulus (A). Grand Mean TFR of the individual, virtual channel, spatially-filtered single trials for control subjects during Predicted presentation of a stimulus. The plot was baselined using the average spectral energy observed in the pre-stimulus period (-100 – 0 ms). (B) Grand Mean TFR of the individual, virtual channel, spatially-filtered single trials for control subjects during Random presentation of a stimulus. (C) Mean TFR differences between conditions. (D) Statistically significant values remaining once condition differences were thresholded to p </= 0.05. [file 1744-9081-4-8-S3.pdf]

# SI ADHD Stimulus Response

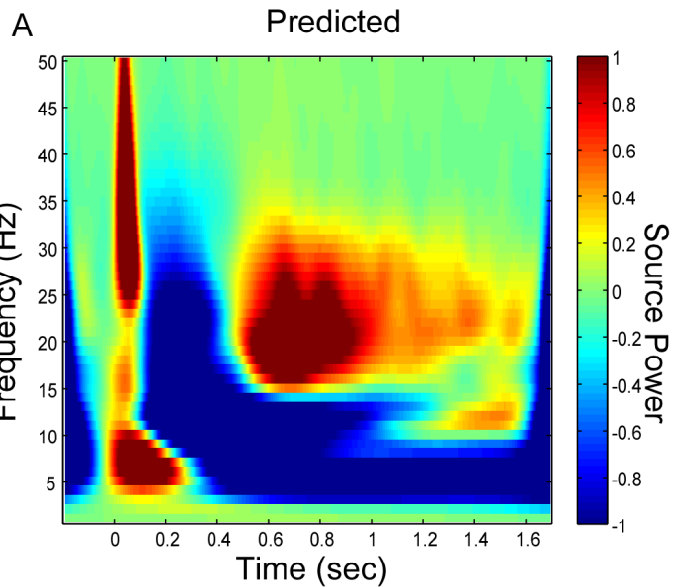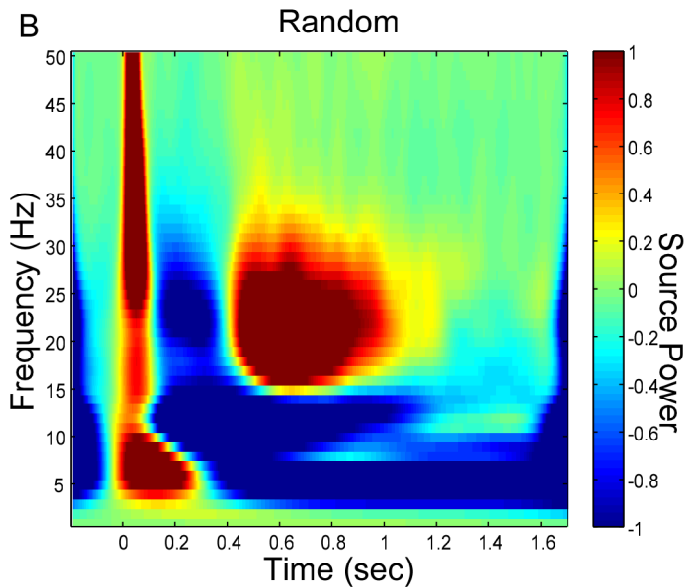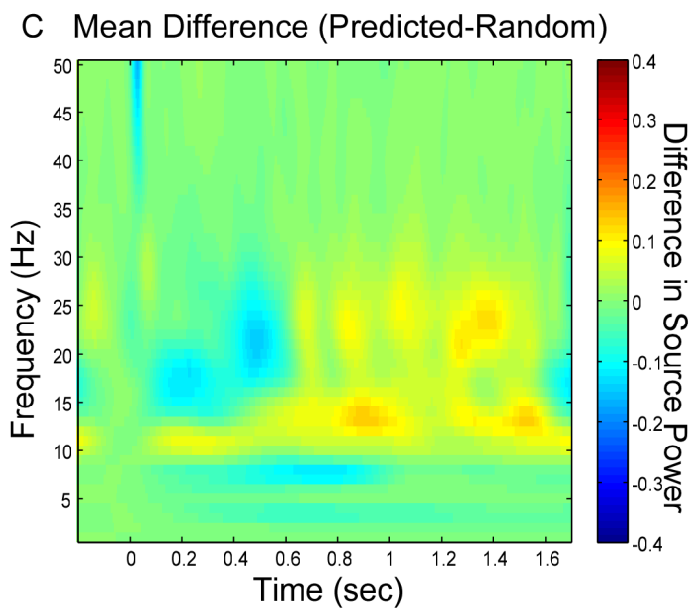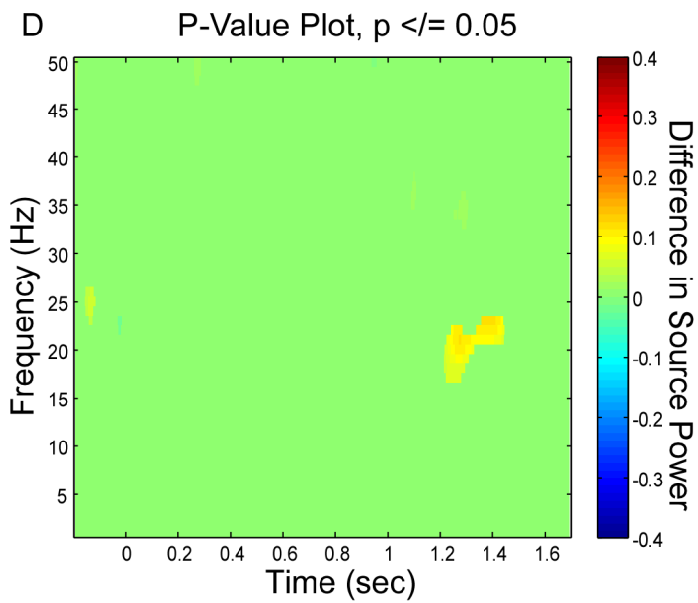

Supplement: Additional file 4 — ADHD SI Frequency and Power Dynamics During Predicted versus Random Presentation of a Somatosensory Stimulus (A). Grand Mean TFR of the individual, virtual channel, spatially-filtered single trials for subjects with ADHD during Predicted presentation of a stimulus. The plot was baselined using the average spectral energy observed in the pre-stimulus period (-100 – 0 ms). (B) Grand Mean TFR of the individual, virtual channel, spatially-filtered single trials for subjects with ADHD during Random presentation of a stimulus. (C) Mean TFR differences between conditions. (D) Statistically significant values remaining once condition differences were thresholded to p </= 0.05. [file 1744-9081-4-8-S4.pdf]

# SII Control Stimulus Response

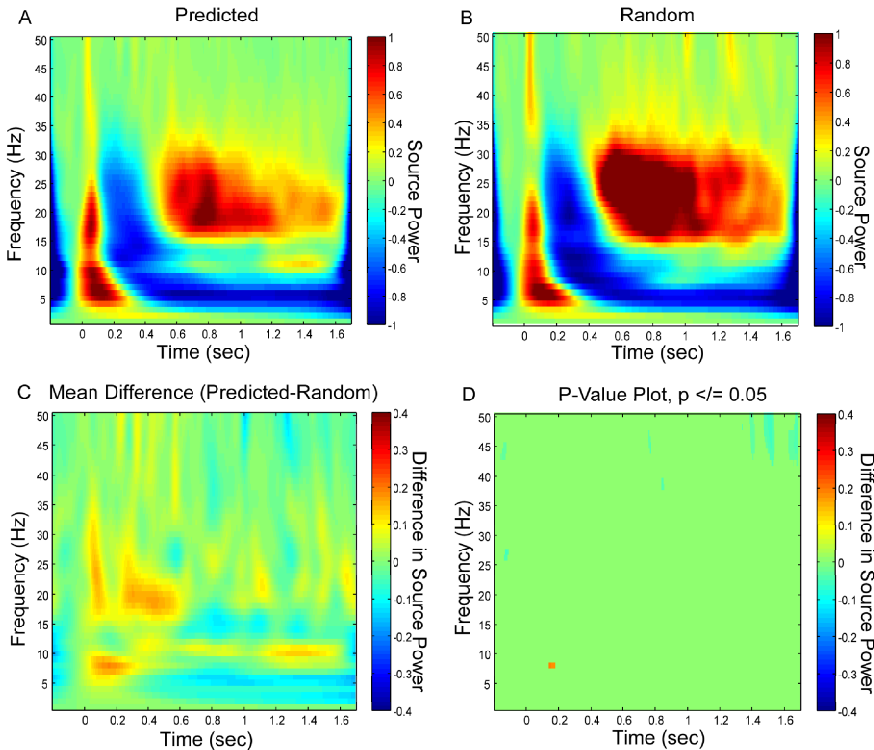

Supplement: Additional file 5 — Control SII Frequency and Power Dynamics During Predicted versus Random Presentation of a Somatosensory Stimulus (A). Grand Mean TFR of the individual, virtual channel, spatially-filtered single trials for control subjects during Predicted presentation of a stimulus. The plot was baselined using the average spectral energy observed in the pre-stimulus period (-100 – 0 ms). (B) Grand Mean TFR of the individual, virtual channel, spatially-filtered single trials for control subjects during Random presentation of a stimulus. (C) Mean TFR differences between conditions. (D) Statistically significant values remaining once condition differences were thresholded to p </= 0.05. [file 1744-9081-4-8-S5.pdf]

# SII ADHD Stimulus Response

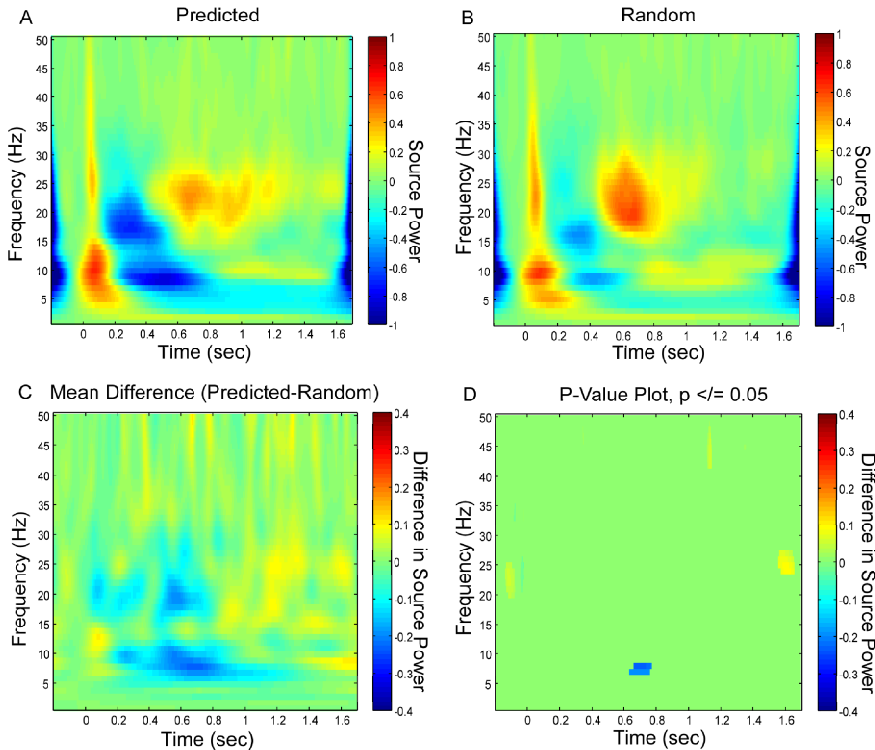

Supplement: Additional file 6 — ADHD SII Frequency and Power Dynamics During Predicted versus Random Presentation of a Somatosensory Stimulus (A). Grand Mean TFR of the individual, virtual channel, spatially-filtered single trials for subjects with ADHD during Predicted presentation of a stimulus. The plot was baselined using the average spectral energy observed in the pre-stimulus period (-100 – 0 ms). (B) Grand Mean TFR of the individual, virtual channel, spatially-filtered single trials for subjects with ADHD during Random presentation of a stimulus. (C) Mean TFR differences between conditions. (D) Statistically significant values remaining once condition differences were thresholded to p </= 0.05. [file 1744-9081-4-8-S6.pdf]
