# Supplementary material for: MEG event-related desynchronization and synchronization deficits during basic somatosensory processing in individuals with ADHD
Source: Behav Brain Funct. 2008 Feb 12;4:8. doi: 10.1186/1744-9081-4-8 (PMC2266931; doi:10.1186/1744-9081-4-8)

ADHD subject

Control Period: -0.2 sec to 0.0 sec　　Active Period: 0.0 sec to 0.2 sec

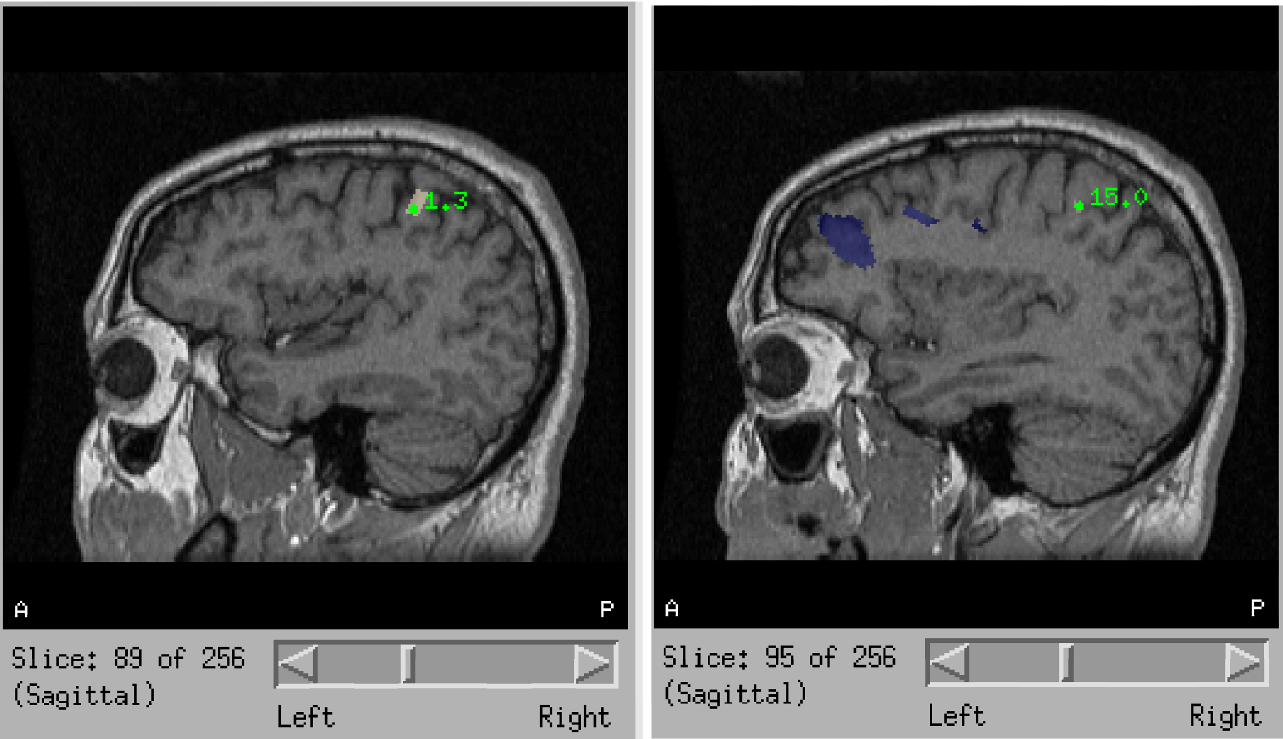

Supplement: Additional file 2 — Example of an ADHD subject's SAM peak locations and values during control (SAM = 1.3) and active (SAM peak value = 15.0) states in somatosensory cortex [file 1744-9081-4-8-S2.pdf]
